# CRISPR-Mediated VHL Knockout Generates an Improved Model for Metastatic Renal Cell Carcinoma

**DOI:** 10.1038/srep29032

**Published:** 2016-06-30

**Authors:** Shiruyeh Schokrpur, Junhui Hu, Diana L. Moughon, Peijun Liu, Lucia C. Lin, Kip Hermann, Serghei Mangul, Wei Guan, Matteo Pellegrini, Hua Xu, Lily Wu

**Affiliations:** 1 Department of Molecular and Medical Pharmacology, David Geffen School of Medicine, University of California at Los Angeles CA 90095, USA; 2 Department of Urology and Institute of Urology, Tongji Hospital, Tongji Medical College, Huazhong University of Science and Technology, Wuhan, 430030, China; 3 Department of Paediatric Surgery, Tongji Hospital, Tongji Medical College, Huazhong University of Science and Technology, Wuhan, 430030, China; 4 Department of Computer Science and Human Genetics, University of California at Los Angeles CA 90095, USA; 5 Department of Molecular, Cell, and Developmental Biology, University of California at Los Angeles CA 90095, USA; 6 Department of Urology, David Geffen School of Medicine, University of California at Los Angeles CA 90095, USA

## Abstract

Metastatic renal cell carcinoma (mRCC) is nearly incurable and accounts for most of the mortality associated with RCC. Von Hippel Lindau (VHL) is a tumour suppressor that is lost in the majority of clear cell RCC (ccRCC) cases. Its role in regulating hypoxia-inducible factors-1α (HIF-1α) and -2α (HIF-2α) is well-studied. Recent work has demonstrated that VHL knock down induces an epithelial-mesenchymal transition (EMT) phenotype. In this study we showed that a CRISPR/Cas9-mediated knock out of VHL in the RENCA model leads to morphologic and molecular changes indicative of EMT, which in turn drives increased metastasis to the lungs. RENCA cells deficient in HIF-1α failed to undergo EMT changes upon VHL knockout. RNA-seq revealed several HIF-1α-regulated genes that are upregulated in our VHL knockout cells and whose overexpression signifies an aggressive form of ccRCC in the cancer genome atlas (TCGA) database. Independent validation in a new clinical dataset confirms the upregulation of these genes in ccRCC samples compared to adjacent normal tissue. Our findings indicate that loss of VHL could be driving tumour cell dissemination through stabilization of HIF-1α in RCC. A better understanding of the mechanisms involved in this phenomenon can guide the search for more effective treatments to combat mRCC.

Kidney and renal pelvis cancers accounted for an estimated 61,650 new cancer cases and 14,080 deaths in 2015^1^. Patients with metastatic disease face a poor prognosis, with a five year survival of less than 12%. Renal cell carcinoma (RCC) makes up 90–95% of these cancers, with the majority of those the clear cell (ccRCC) histological subtype^2^,^3^. Treatment options for metastatic RCC (mRCC) are limited because this tumour shows resistance to traditional chemotherapy and radiation. The one treatment that has cured this condition is interleukin-2 (IL-2) therapy, but only in around 7% of patients^4^. Recent developments of targeted therapies, including those targeting immune checkpoint inhibitor programmed cell death-1 (PD-1), have shown modest efficacy^5^,^6^. The lack of enduring interventions to combat mRCC underscores the need for models that better recapitulate the disease and new insights into the mechanisms driving this condition.

Much of our understanding of ccRCC comes from studies on the tumour suppressor von Hippel Lindau (VHL). Hereditary cases of VHL syndrome show increased risk of ccRCC development^7^,^8^,^9^. Subsequent studies revealed that this gene is also silenced in up to 90% of sporadic ccRCC cases^10^. VHL’s best-described role involves its regulation of the hypoxia response through its recognition and targeting of the alpha subunits of hypoxia-inducible factor (HIF-1α, HIF-2α and HIF-3α) for ubiquitination and degradation^11^,^12^,^13^,^14^,^15^,^16^. In low oxygen conditions, VHL cannot recognize the HIF-αs and they combine with HIF-1β to translocate to the nucleus and enact the transcriptional program necessary for the hypoxic response^17^,^18^.

Researchers have attempted to derive murine models of ccRCC by targeting VHL for knockout^19^,^20^,^21^,^22^,^23^. Recent work has demonstrated that loss of Bap1 in addition to VHL may aid in modelling ccRCC in mice more consistently^24^. Though some of these studies show signs of early cystic ccRCC changes and local neoplasms, they all fail to produce an aggressive, metastatic form of this disease. For this reason, many studies depend on the RENCA model, the most widely used immunocompetent murine model of RCC^25^,^26^,^27^,^28^. This line was isolated from a spontaneously arising tumour in a BALB/c mouse in 1973^29^. When implanted under the kidney capsule, this tumour metastasizes to sites seen in clinical ccRCC, including the lungs, liver and lymph nodes^30^. Despite the proven utility of this murine model, a major concern of its clinical applicability involves its expression of wild type VHL.

Previous work indicates that VHL loss may promote a more aggressive and metastatic tumour model. A number of studies have shown that targeting VHL function can lead to elements of epithelial-mesenchymal transition (EMT)^31^,^32^,^33^. This process has been identified as a central node through which carcinomas must pass to spread from their primary site to other parts of the body^34^. EMT involves the loss of cell-cell contact and a breaking away from the basement membrane of epithelial cells as they transition toward a more migratory and invasive cell type^35^. Concurrent with these phenotypic changes are an assortment of molecular changes, including loss of epithelial markers such as E-cadherin, a common occurrence in clinical ccRCC specimens^31^,^36^, and gain of mesenchymal markers such as N-cadherin and alpha smooth muscle actin (α-SMA)^37^. Notably, a number of studies demonstrate the role of HIF-1α in driving these changes^32^,^33^,^38^. Additionally, HIF-1α has been shown to cause metastasis in other tumour models^38^,^39^. These findings indicate that VHL deletion in the RENCA model may produce a more metastatic, clinically relevant model.

The clustered regularly interspaced short palindromic repeat (CRISPR) method of genetic manipulation has recently been harnessed for routine lab studies^40^. This breakthrough technique of gene disruption is notable for its ease of use and effectiveness in completely knocking out gene function. Based on the *Streptococcus pyogenes* adaptive immune system, this RNA-based technique for genome editing has quickly proved its utility in a number of biological studies^41^. Researchers have developed CRISPR methods in order to generate knockout mice, do genome-wide screens in cell lines, knock out genes in mice *in vivo* and screen for metastatic genes *in vivo*^42^,^43^,^44^,^45^,^46^,^47^. However, few studies thus far demonstrate CRISPR-mediated modification of murine cell lines in order to generate improved transplantable murine cancer models.

In this study, we utilized the new CRISPR genome editing tool to knock out VHL expression in RENCA cells. We show that this change produced increased aggressive behaviour *in vitro* and increased metastasis *in vivo*. This effect depended on HIF-1α, as knockout of this gene in VHL null cells reversed the noted phenotypic and molecular changes consistent with EMT. We went on to identify a set of four HIF-1α-regulated genes that are overexpressed in clinical RCC samples and are associated with poorer survival according to the TCGA database.

## Results

### Generation of VHL knockout RENCA cells using CRISPR

We obtained the lentiCRISPR plasmid developed by the Zhang lab^44^ and generated LCGFP, which has enhanced green fluorescent protein (eGFP) in place of the puromycin resistance gene in lentiCRISPR. Using the CRISPR design tool (crispr.mit.edu), we found candidate gRNA sequences targeting murine VHL. gRNAs against *Renilla* luciferase were also generated to serve as vector controls (Supplementary Fig. S1a and Table S1). RENCA FLuc cells, which stably express firefly luciferase, were transduced with either one of the VHL gRNAs or two. VHL gene sequencing provides an example confirming the specific ten base pair deletion in a region targeted by gRNA1 (g1) (Supplementary Fig. S1b). Protein levels of HIF-1α and expression of its downstream target Glut-1 indicated that g1 was more potent than gRNA2 (g2), given equal expression of eGFP as a proxy for transduction delivery load (Supplementary Fig. S1c–e). Notably, the maximum efficiency of VHL knockout was when both guides were used together. For this reason, we took a two target approach in the generation of the VHL knockout (RVN) and control (RC) cell lines (Supplementary Table S2).

**Figure 1:**
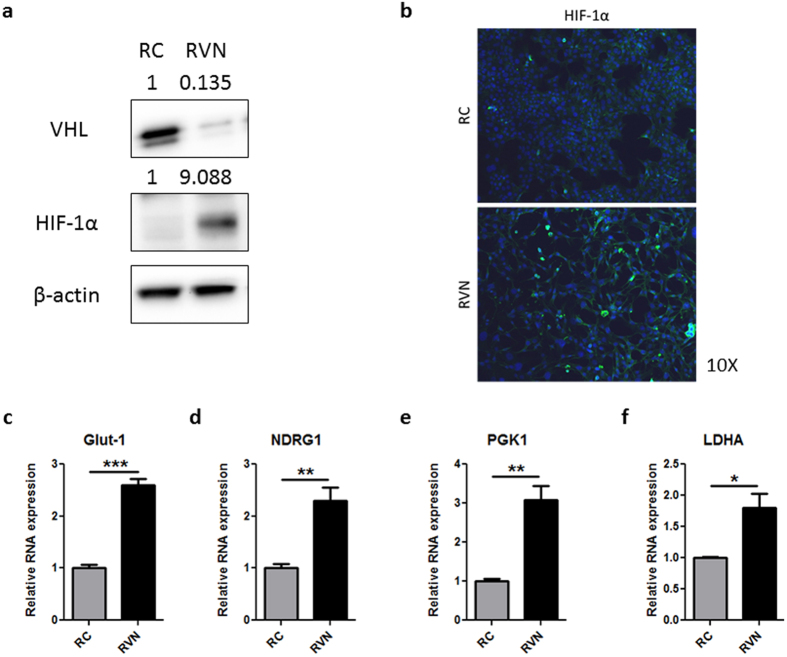
CRISPR-mediated VHL knockout upregulates HIF pathways. (**a**) Western blot for murine VHL, HIF-1α and β-actin in RC and RVN cells. Normalized quantification by densitometry shown above blot. (**b**) Immunofluorescent staining for HIF-1α in RC (top) and RVN (bot.) cells. Nuclei are stained blue with DAPI. Gene expression was analysed by RT-PCR for (**c**) Glut-1, (**d**) NDRG1, (**e**) PGK1 and (**f**) LDHA. n = 3 for RT-PCR studies *denotes p < 0.05, **denotes p < 0.01, ***denotes p < 0.001.

RC and RVN cells were assessed for VHL protein levels to determine the effectiveness of VHL knockout. Western blots revealed a dramatic reduction in VHL upon CRISPR-mediated gene modification (Fig. 1a). Levels of HIF-1α protein were greatly increased in the RVN cells, a change that is expected given that VHL degrades HIF-1α. Since the HIFs are transcription factors, activation of these proteins would be expected to induce nuclear localization. Immunofluorescent staining for HIF-1α in RVN cells revealed increased punctate staining, suggestive of nuclear localization, compared to RC cells (Fig. 1b). Elevation of HIF target genes such as Glut-1, N-myc downstream regulated gene 1 (NDRG1), phosphoglycerate kinase 1 (PGK1) and lactate dehydrogenase A (LDHA), were significantly increased in RVN compared to RC cells (Fig. 1c–f). Notably, Glut-1 and NDRG1 have been shown to be regulated by both HIF-1α and HIF-2α, whereas PGK1 and LDHA are HIF-1α-specific^48^. These findings indicate that this CRISPR approach can effectively knock out VHL, and that its well-studied role in modulating the HIF pathways is intact in RENCA cells.

### Loss of VHL induces EMT with enhanced aggressive *in vitro* behaviour

**Figure 2:**
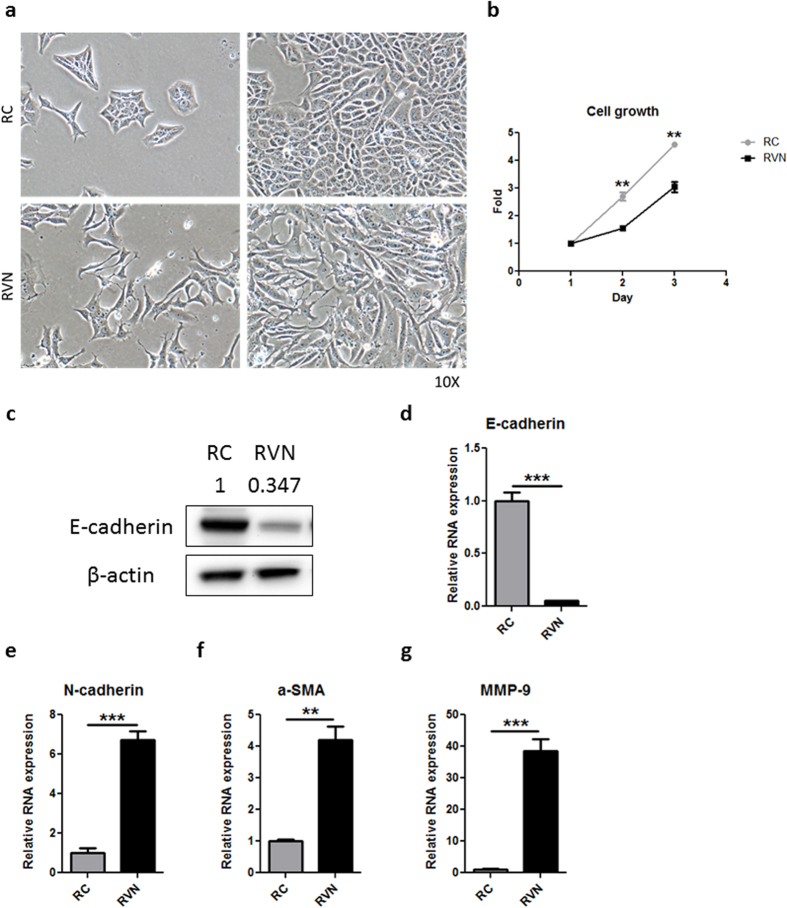
VHL loss induces morphologic and molecular changes indicative of EMT. (**a**) Phase contrast 10X images of RC and RVN cells at low (left) and high (right) densities. (**b**) Growth chart of RC versus RVN cells. (**c**) Western blot for murine E-cadherin and β-actin in RC and RVN cells. Normalized quantification by densitometry shown above blot. Gene expression was analysed by RT-PCR for (**d**) E-cadherin, (**e**) N-cadherin, (**f**) α-SMA and g) MMP-9. n = 3 for proliferation assay and RT-PCR, **denotes p < 0.01, ***denotes p < 0.001.

CRISPR-mediated VHL knockout induced a dramatic phenotypic change in RVN cells that was readily observable when compared to RC cells. While RC cells demonstrated the typical cobblestone epithelial morphology like wild type RENCA cells (Fig. 2a, top, Supplementary Video S1), RVN cells adopted an elongated, fibroblastic morphology with reduced cell-cell contacts (Fig. 2a, bottom, Supplementary Video S2). Interestingly, RVN cells had reduced proliferation when compared to RC cells (Fig. 2b). The morphology and proliferation changes were consistent with RVN cells undergoing EMT. For this reason, we chose to investigate whether VHL loss might also be driving EMT. Protein and RNA levels of E-cadherin, expected to be down following EMT, were reduced in RVN cells (Fig. 2c,d). Genes associated with the mesenchymal phenotype, such as N-cadherin, α-SMA and matrix metalloproteinase 9 (MMP-9), were significantly upregulated in RVN cells when compared to RC cells (Fig. 2e–g).

Questions can be raised regarding the ability to attribute the molecular changes in our cells to loss of VHL and not a non-specific effect. For these reasons, we grew out clonal cell lines from RENCA cells transduced with RLuc g2, mVHL g1 and mVHL g3 (Supplementary Tables S1 and S2). Multiple gRNAs targeting distinct portions of VHL produced the expected VHL gene knockout and molecular phenotype consistent with RVN cells (Supplementary Fig. S2a–e). Notably, the gRNA targeting *Renilla* luciferase failed to produce these effects. Furthermore, rescue experiments using a doxycycline-regulated system showed that re-expression of VHL in a RENCA VHL knockout cell line leads to a reversion to an epithelial morphology (Supplementary Fig. S2f,g). Taken together, these findings demonstrate that our EMT changes are due to VHL loss and not through a non-specific effect caused by the CRISPR system or gRNAs.

Previous studies have demonstrated HIF-1α stabilization and EMT changes in human VHL wild-type RCC cell lines upon VHL knockdown^33^,^49^. We generated ACHN VHLko cells using gRNAs that targeted regions of human VHL that were orthologous to the sequences that were targeted in murine VHL to create the RVN cell lines (Supplementary Table S1). ACHN control cells were transduced with a gRNA targeting *Renilla* luciferase. As expected, CRISPR-mediated targeting of VHL led to a reduction in VHL protein levels and an increase in HIF-1α (Supplementary Fig. S3a). Morphologic changes in ACHN VHLko cells were consistent with those seen in RVN cells (Supplementary Fig. S3b). Molecular changes in ACHN VHLko were also as expected, with a reduction in E-cadherin and increased MMP9 expression, consistent with two separate gRNAs (Supplementary Fig. S3c,d).

**Figure 3:**
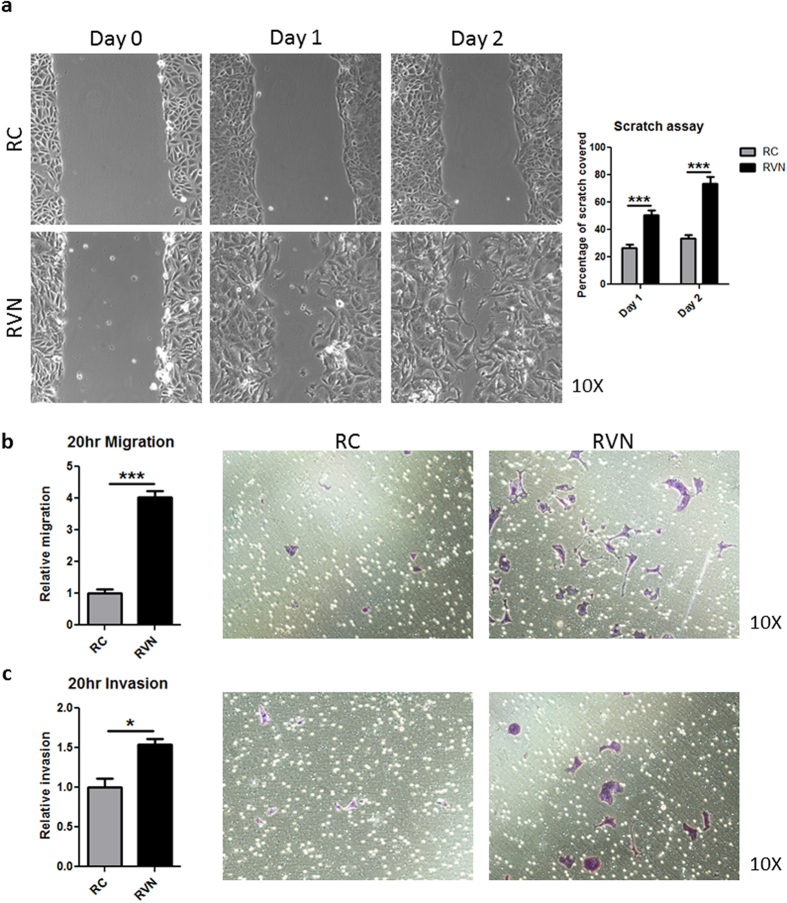
VHL knockout induces increased RENCA cell migration and invasion *in vitro*. (**a**) Images of a scratch assay were taken at the same location one and two days after scratching. Representative images of each cell type are shown (left) and quantification of scratch filling is shown to the right. (**b**) Quantification (left) and representative images (right) of RC and RVN migration are shown. (**c**) Quantification (left) and representative images (right) of RC and RVN invasion are shown. n = 7 for scratch assay, n = 3 for migration and invasion assays, *denotes p < 0.05, ***denotes p < 0.001.

Given that the morphological and molecular changes in RVN cells were consistent with an EMT, we set out to determine whether these translated to a more aggressive phenotype. We performed a scratch assay and observed the patterns of scratch resolution for the two cell types. While RC cells resolved the scratch as a connected sheet, RVN cells moved into the open area individually (Supplementary Videos S3 and S4). We quantified the resolution of the scratch over two consecutive days (Fig. 3a). At day 1, RC cells had resolved 26.34% (+/−2.619) of the scratch compared to 50.54% (+/−3.372) for RVN cells. At day 2, RC cells had covered 33.56% (+/−2.642), while RVN cells resolved 73.57% (+/−5.227). These findings suggested a dramatically increased migratory capacity for RVN cells. Confirming this phenotype, a transwell assay showed RVN cell migration was four times greater than RC cells (Fig. 3b), and invasion of RVN cells through a matrigel transwell was more than 1.5 times that of RC cells (Fig. 3c). These findings suggest that RVN cells may have a greater metastatic potential.

### VHL knockout drives increased metastasis

**Figure 4:**
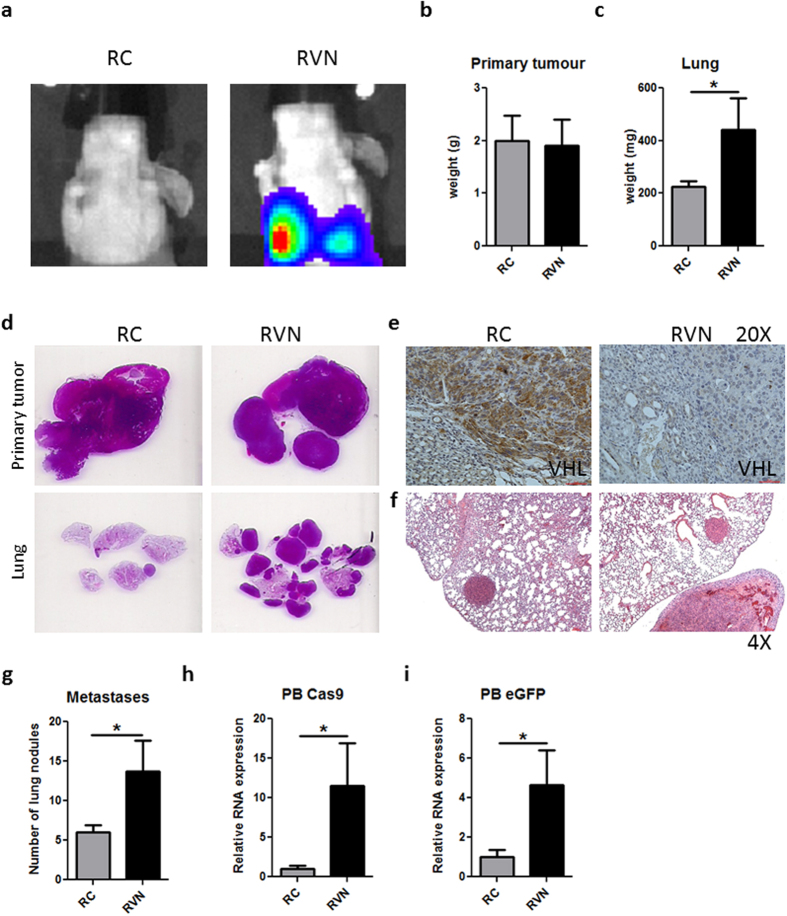
Metastasis from orthotopic site is enhanced by VHL knockout. RC and RVN cells were implanted under the kidney capsule of Nu/J mice. Animals were sacrificed at four weeks following implantation. (**a**) Representative bioluminescent images at day of endpoint are shown. Primary tumours (**b**) and lungs (**c**) were weighed and quantified for the two groups. (**d**) H&E stains are shown from primary tumour and whole lung. (**e**) Immunostaining for VHL in representative RC and RVN primary tumours shown at 20X. (**f**) 4X H&E representative images of RC and RVN lung nodules. (**g**) Lung nodules were counted and quantified. Gene expression of Cas9 (**h**) and eGFP (**i**) in the peripheral blood was assessed as a measure of circulating tumour cells. n = 3–4 animals per group for weights and RT-PCR, n = 6–8 per group for lung nodule counts *denotes p < 0.05.

Following our demonstration of the increased aggressive phenotype of RVN cells *in vitro*, we sought to evaluate their ability to metastasize from a primary orthotopic tumour. Attempts to establish our RC and RVN tumours in immunocompetent BALB/c mice yielded inconsistent results characterized by failure to establish, or even rejection of, the orthotopic and subcutaneous tumours. We surmised that an immune-mediated rejection directed against CRISPR components could be at play as the use of transient expressing, non-integrating lentiCRISPR overcame this problem (data not shown). Thus, we decided to implant RC and RVN orthotopically in Nu/J mice. Animals were bioluminescently imaged four weeks post-injection and increased lung signal was revealed in RVN mice (Fig. 4a). While primary tumour size was similar in both groups, the lungs of mice implanted with RVN cells weighed significantly more (Fig. 4b,c). H&E confirmed comparable primary tumour sizes but increased lung metastasis (Fig. 4d), VHL knockout in primary tumours and increased metastatic burden in the lungs (Fig. 4e,f). H&E slides of lungs revealed significantly more lung metastases in RVN-implanted mice when compared to RC-implanted mice (Fig. 4g). Using RT-PCR for Cas9 and eGFP, circulating tumour cells were significantly increased in the peripheral blood of animals implanted with cells deficient in VHL (Fig. 4h–i).

### VHL loss induces HIF-1α-mediated EMT

**Figure 5:**
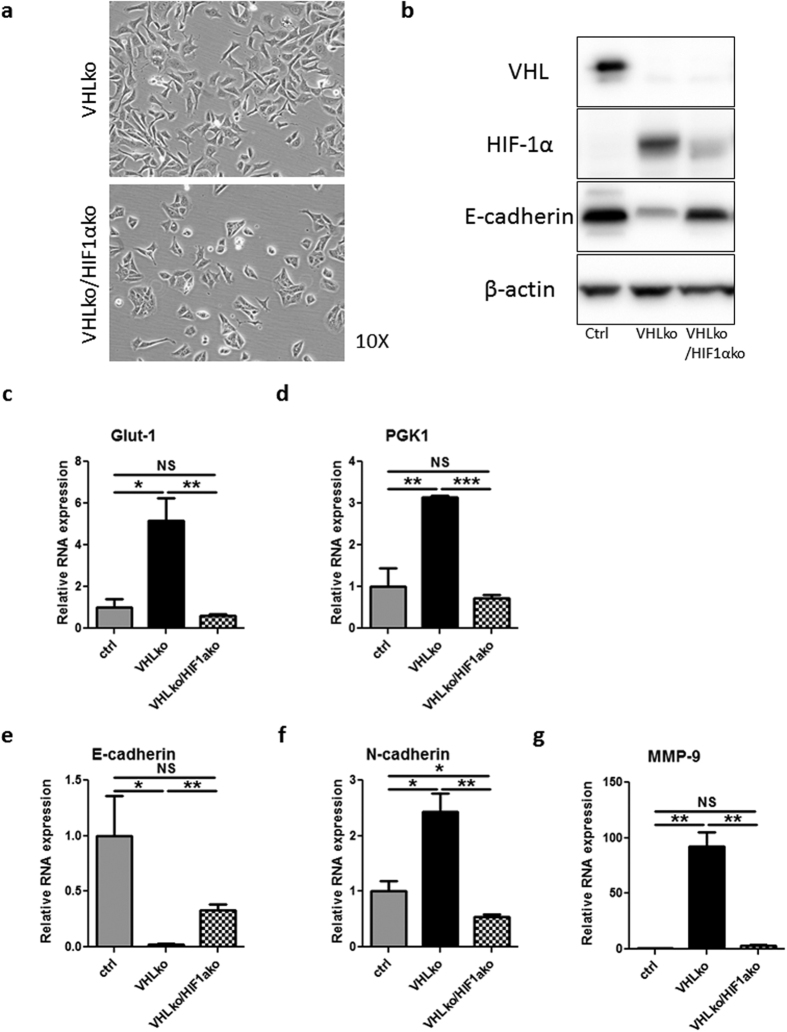
CRISPR-mediated knockout of HIF-1α in VHL knockout clonal cell line shows morphological and molecular reversion of EMT. The V1c1 RENCA clonal cell line was transduced with LCGFP RLuc g1 (VHL KO) or LCGFP mHIF-1α g1 (VHLko/HIF1ako). Ctrl cells are the Rc1 clonal cell line. (**a**) Phase contrast 10X images of VHLko and VHLko/HIF-1αko cells. (**b**) Western blot for murine VHL, HIF-1α, E-cadherin and β-actin. Gene expression was analysed by RT-PCR for (**c**) Glut-1, (**d**) PGK1, (**e**) E-cadherin, (**f**) N-cadherin and (**g**) MMP-9. n = 3 for RT-PCR studies *denotes p < 0.05, **denotes p < 0.01, ***denotes p < 0.001, NS denotes no significance.

We began to interrogate the mechanism responsible for VHL-mediated EMT in RENCA cells. Previous work has implicated HIF-1α in the EMT process in RCC cells. To explore HIF-1α’s role in driving EMT in our cells, we used CRISPR to target HIF-1α or *Renilla* Luciferase in the V1c1 clonal VHL knockout cell line (VHLko/HIF1αko and VHLko, respectively, Supplementary Table S2). For a control, we used the clonal line targeting only *Renilla* luciferase (Rc1, Supplementary Table S2). The mesenchymal morphology exhibited by VHLko cells was reverted back to an epithelial appearance in VHLko/HIF1ako (Fig. 5a). HIF-1α loss in VHLko cells led to re-expression of E-cadherin (Fig. 5b,e), significantly decreased Glut-1 and PGK1 RNA expression (Fig. 5c,d), and increased expression of N-cadherin and MMP-9 (Fig. 5f,g). These morphological and molecular changes were consistent with HIF-1α mediation of VHL knockout-induced EMT. Similar findings were obtained with a VHL and HIF-1α double knockout from wildtype, non-clonal RENCA cells (Supplementary Fig. S4). It is unlikely that HIF-2α contributes to this EMT phenotype given its low level of expression compared to HIF-1α in these cells (Supplementary Fig. S5a). Furthermore, a HIF-2α small molecule inhibitor (N-(3-chloro-5-fluorophenyl)-4-nitrobenzo[c][1,2,5]oxadiazol-5-amine) was unable to reverse the phenotype or molecular changes of VHL knockout despite downregulation of HIF-2α-responsive genes erythropoietin (EPO) and vascular endothelial growth factor A (VEGF-A) (Supplementary Fig. S5b,c).

To further confirm the hypoxia-mediated EMT phenotype, we used Cobalt (II) chloride (CoCl_2_) and Dimethyloxalylglycine, N-(Methoxyoxoacetyl)-glycine methyl ester (DMOG), two hypoxia mimetic chemicals that do not affect VHL but stabilize HIFs. Treatment with either of these chemicals induced morphologic changes indicative of EMT in RENCA cells following one week of treatment (Supplementary Fig. S6a). Of interest, the VHL knockout-induced EMT appears to be independent of EMT-promoting transcription factors such Snail1/2, Twist, Zeb1 and Zeb2, as the expression levels of these genes are not elevated in RVN compared to RC cells (Supplementary Fig. S6b). Taken together with our previous findings, we can conclude that the EMT observed in VHL knockout cells can be attributed to HIF-1α.

### VHL loss leads to HIF-1α-mediated upregulation of genes representative of aggressive clinical ccRCC

**Figure 6:**
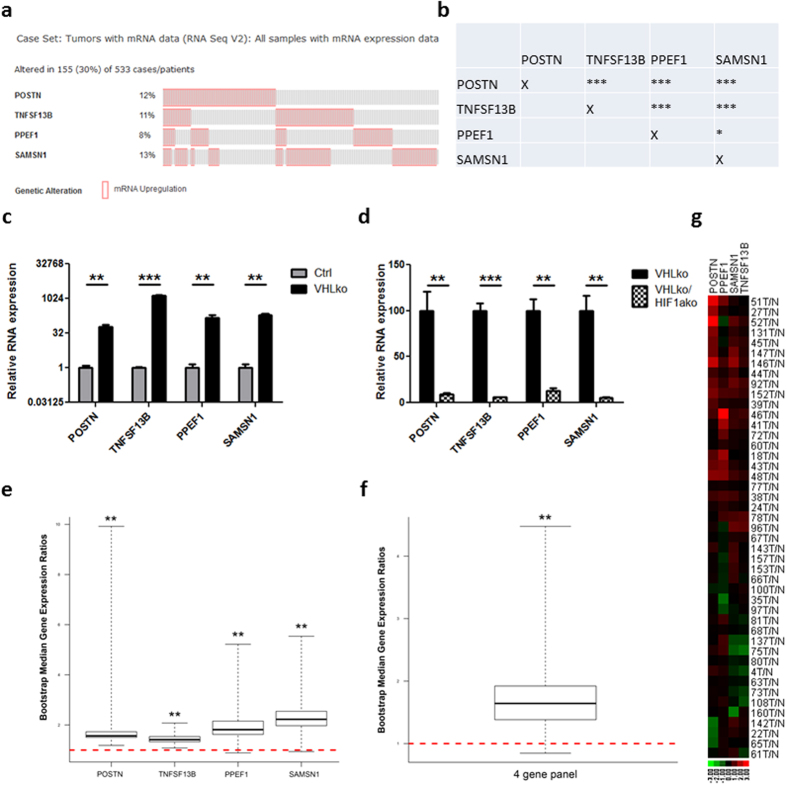
Four-gene signature shows frequent co-occurrence and upregulation in ccRCC samples. (**a**) Frequency (%) of having upregulation one standard deviation higher than the mean for each of the four genes in the TCGA ccRCC database is given (left of each row). Each column represents one individual patient. Data for all patients with at least one upregulated gene is shown. (**b**) Table of co-occurrence analysis for the four genes. Gene expression was analysed by RT-PCR for POSTN, TNFSF13B, PPEF1 and SAMSN1 comparing (**c**) Ctrl and VHLko RENCA cells and (**d**) VHLko and VHLkoHIF1ako RENCA cells. Expression of these four genes was assessed in 45 clinical ccRCC samples from radical nephrectomies. Comparison of gene expression between tumour and normal adjacent tissue was analysed BootstRatio method for (**e**) each individual gene and (**f**) the 4 gene panel. (**g**) Heat map demonstrating expression of these four genes in each of the 45 clinical ccRCC samples. *denotes p < 0.05 **denotes p < 0.01 ***denotes p < 0.001.

Findings from the RVN model have thus far implicated HIF-1α as a key pathway driving RCC dissemination. HIF-1α has already been associated with more aggressive ccRCC, and shown to drive metastasis in other tumour models^38^,^39^,^50^. However, the molecular pathways downstream of HIF-1α that modulate RCC metastatic behaviours are poorly understood. The RC and RVN cells were submitted for RNA-seq and over 8,500 genes were found to be differentially expressed between the two cell lines. Each of the top 65 upregulated genes in the RNA-Seq data set were queried with the TCGA data set, leading to a list of about ten genes that were individually indicative of a significantly poorer overall and/or progression free survival (Supplementary Table S3). Remarkably, four of these genes, of which 30% percent of patients had upregulation of one or more, seemed to be correlated in expression. These include periostin (POSTN), TNFSF13B (also known as B-cell-activating factor or BAFF), PPEF1 and SAMSN1 (Fig. 6a,b). Of note, these genes are all HIF-1α regulated, as evidenced by their induction with VHL knockout (Fig. 6c) and downregulation upon HIF-1α loss (Fig. 6d).

To further verify that the upregulation of these four genes is indeed relevant in clinical ccRCC, we analysed gene expression in an independent set of clinical samples. We utilized 45 ccRCC tumour samples, recently collected from radical nephrectomies, and compared the expression of these four genes to adjacent normal tissue. POSTN, PPEF1, SAMSN1 and TNFSF13B were upregulated in a great majority of the ccRCC tumours (Supplementary Fig. S7a–d). Analysed by the BootstRatio method^51^, the upregulation of each gene was statistically significant (Fig. 6e, POSTN: Median.Ratio.Obs = 1.567, p < 0.01; PPEF1: Median.Ratio.Obs = 1.8139, p < 0.01; SAMSN1: Median.Ratio.Obs = 2.2257, p < 0.01; TNFSF13B: Median.Ratio.Obs = 1.4153, p < 0.01). Analysis of the geometric mean of 4 genes also showed a statistically significant upregulation in the tumours (Fig. 6f) (Median.Ratio.Obs = 1.6423, p < 0.01). In addition, we performed a log transformation with base 10 of the fold change, in which red indicates upregulation and green indicates downregulation (Fig. 6g). In this heat map, it is clear that a majority of the tumours from the 45 ccRCC patients have an upregulated pattern in this four gene set.

**Figure 7:**
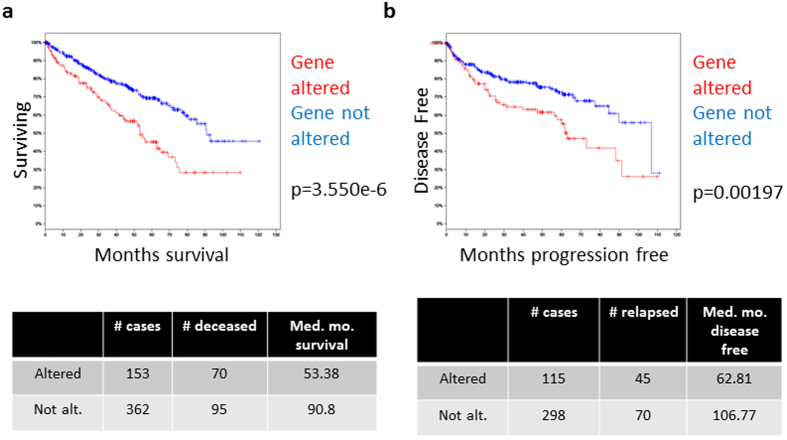
Four-gene signature predicts poor clinical outcomes in ccRCC patients. Kaplan-Meier curves of (**a**) overall survival and (**b**) disease-free survival for the patients with upregulation of one or more of POSTN, TNFSF13B, PPEF1 and SAMSN1 is compared to patients without upregulation of any of these genes in the TCGA.

We again utilized the TCGA database to glimpse the impact of the upregulation of these 4 genes on the outcome in patients. The median overall survival for patients with upregulation of one or more of these genes was 53.38 months, compared to 90.8 months for patients without upregulation (p = 3.550e–6, Fig. 7a). Similarly, median progression-free survival for patients with gene upregulation was 62.81 months compared to 106.77 months (p = 0.00197, Fig. 7b). Collectively, our interrogation into clinical sources clearly showed that the four biomarker genes derived from our RVN model are upregulated in the ccRCC tumour tissues and that they predict a significantly poorer outcome in patients with this disease.

## Discussion

This study demonstrates the ability to use a lentiviral CRISPR approach to manipulate a murine cell line *in vitro* and generate an improved mRCC tumour model *in vitro* and *in vivo*. There are a number of noteworthy aspects of the RENCA VHL null model we generated. Since VHL loss is the central hallmark of ccRCC clinically, we have now made the historically used RENCA model more genetically similar to what is seen in patients. The resulting dramatic morphologic and molecular changes, such as the E-cadherin loss that is commonly seen in the clinic^36^, indicates that our new ccRCC model is more similar and applicable to clinical disease. Characterizing the differences in tumour progression between RENCA VHL null cells and RENCA control cells should be the focus of future studies. We expect that these differences will reveal insights into key aspects of ccRCC.

Preliminary analysis of our cell lines indicates that many genes that correspond with a poor clinical prognosis in ccRCC patients are upregulated in the RENCA VHL null cells. We describe four genes that are HIF-1α regulated, frequently co-expressed and associated with poor survival clinically. Our independent validation of these genes showed that they are statistically significantly upregulated in ccRCC compared to adjacent normal tissue, with two or more frequently being upregulated concurrently. Interestingly, several of these genes have been tied to aggressiveness in other types of cancers. Periostin is involved in cell adhesion and motility, and some studies have shown its expression is associated with poor survival in a number of cancers, including RCC^52^,^53^. It has been recently demonstrated that periostin is associated with metastasis in head and neck cancer, and that this protein directly accelerated the growth, migration and invasion of cancer cells^54^. Another group found that periostin was overexpressed in a clinical case of melanoma metastasis and subsequently showed that inhibition of this protein in stromal cells leads to enhanced adhesion and reduced metastatic spread in a murine melanoma model^55^. TNFSF13B enhanced cell motility and invasion in a study of pancreatic cancer^56^. SAMSN1 was found to be expressed highly in glioblastoma multiforme, and high expression was a significant risk factor for poor survival^57^. Evaluating these genes and PPEF1, a serine/threonine protein phosphatase of which little is known, may produce potentially novel predictors of ccRCC prognosis. Additionally, they might provide new, clinically meaningful targets for aggressive ccRCC. Our findings warrant further validation of these markers with regards to ccRCC and an investigation into their potential mechanisms for promoting tumour aggressiveness in kidney cancer.

The role of HIF-1α in driving much of the aggressive phenotype in the RENCA VHL null cells is a noteworthy and intriguing finding of our studies. Though HIF-1α was originally thought to play a major part in ccRCC progression, recent studies have questioned this role^58^,^59^. Current thinking suggests that HIF-1α acts as a tumour suppressor, slowing growth of ccRCC cells^60^. Our findings showing reduced growth rate *in vitro* of RENCA VHL knockout cells are in line with this view. However, our results suggest that while HIF-1α may reduce proliferation, this may come with the caveat of increased migratory and invasive capacity. This agrees with the “Grow” or “Go” hypothesis, which argues that cells choose between proliferation and mobility and do not participate in both at once^61^. Some investigators have shown that HIF-1α inhibits primary tumour growth, whereas HIF-2α enhances it^60^. Combined with our findings, this suggests that VHL loss creates an environment where HIF-1α expression drives tumour metastasis and HIF-2α promotes tumour growth. There is evidence that HIF-1α expression correlates with worse prognosis for metastatic ccRCC patients^50^, and our study identifies some potential HIF-1α-driven genes that could contribute to a more aggressive ccRCC phenotype. Clarification of the differential roles of HIF-1α and HIF-2α with regard to metastasis promotion is critical for better understanding of this disease process and development of effective interventions.

The role of EMT in driving metastasis is a topic of great interest and pertinent in this new model for RCC metastasis. Recent work has suggested that EMT is not necessary for metastatic spread but may play a role in resistance to chemotherapy^62^,^63^. Notably, in our model, it is unclear whether the increased metastatic outgrowth we see in the VHL knockout model is through direct spread of the mesenchymal cells that have undergone the EMT. While this may be at play in our model, several other factors secondary to the mesenchymal changes in the cells could promote metastatic spread of neighbouring tumour cells. Upregulation of genes such as POSTN, TNFSF13B and SAMSN1 could provide increased metastatic potential of the tumour, as discussed. Additionally, other factors, such as CXCL1 and CXCL2 are significantly elevated with loss of VHL (data not shown) and could promote metastasis through a number of paracrine mechanisms^64^,^65^. Whether the EMT in this model leads to a direct or indirect promotion of metastasis will be the focus of future investigation.

A major question that arises from our findings involves the timing of VHL loss and how it impacts tumour behaviour. The RENCA cell line represents an already transformed tumour. We find that disrupting VHL on this background creates a more aggressive phenotype. One may wonder how well this sequence represents clinical disease since patients are thought to lose VHL early in the development of ccRCC^66^. It is possible that, for aggressive ccRCC, what matters is not the order in which VHL is lost, but the pathways that are disrupted in addition to its loss. The transforming mutations that drive the RENCA cell line remain elusive, and determining the molecular changes responsible for RENCA tumorigenesis should receive some attention in future studies. Once these are known, CRISPR technologies can be used to generate RCC tumours *in vivo* that will better model ccRCC. These models will enhance our understanding of the progression of ccRCC and could potentially provide insights into novel targeted therapies that could prove more effective in combating mRCC. This study is the first to use CRISPR-mediated technologies to explore the biology of VHL loss driving RCC aggressiveness in a new murine model and has produced intriguing clues regarding the involvement of HIF-1α in metastatic dissemination, new potential marker genes to assess poor prognosis of clinical ccRCC and possible molecular targets to combat mRCC aggressiveness.

## Methods

### Generation of lentiCRISPR-eGFP (LCGFP) and CRISPR cloning

LentiCRISPR (LC, Addgene #49535) and pX458 (Addgene #48138) were gifts from Dr. Feng Zhang^67^,^68^. LC was digested with NheI and MluI. A region of pX458 was amplified by PCR to produce NheI-2A-eGFP-MluI. DNA fragments ligated using Quick Ligation Kit (NE Biolabs). gRNA sequences for murine and human VHL and HIF-1α were produced by the CRISPR design tool software^69^ and oligonucleotides ordered from Valuegene. gRNAs were cloned into LC and LCGFP as detailed in the Zhang lab protocol^70^,^71^. gRNA sequences are listed in Supplementary Table S1. Cell lines are listed in Supplementary Table S2.

### Cell culture and proliferation assay

RENCA and ACHN (ATCC) were cultured in Dulbecco’s Modification of Eagle’s Medium (DMEM) supplemented with 10% foetal bovine serum (FBS), 100 U/ml penicillin and 100 ug/ml streptomycin. RENCA cells were transduced to express firefly luciferase to generate the RENCA FLuc line as previously described^72^. All cell incubations were carried out at 37 °C and at 5% CO_2_. Puromycin selection was performed at 2 ug/ml for 5–7 days. Clonal selections were performed with Bel-Art Scienceware cloning discs according to manufacturer’s instructions. To analyse for proliferation, cells were plated in 6 well dishes at 1 × 10 ^ 5 cells per well. Each day, on three consecutive days, cells were trypsinized and counted using a Vi-CELL counter (Beckman Coulter).

### Western blot, immunofluorescence and RT-PCR to analyse gene expression

Cell lysates were obtained, resolved on gels and transferred as previously described^73^. Blots were probed with antibodies recognizing VHL (Santa Cruz Biotechnology FL-181, 1:200), HIF-1α (Novus Biologicals H1alpha67, 1:500), E-cadherin (BD Biosciences 36/E-cadherin, 1:10,000) and β-actin (Santa Cruz Biotechnology C4, 1:5,000). Blots were imaged and densitometry performed on a ChemiDoc XRS + with associated ImageLab software (Bio-Rad).

For immunofluorescence staining, 5 × 10 ^ 5 cells per well were plated onto gelatine-coated cover glasses in a 12-well dish. Cells were fixed and stained with antibodies recognizing HIF-1α (Novus Biologicals H1alpha67, 1:100) and HIF-2α (Novus Biologicals ep190b, 1:100). Nuclei were stained with DAPI during mounting using Prolong Gold antifade mounting reagent (Life Technologies). Quantitative RT-PCR gene expression studies were conducted as previously described^74^. Primer sequences can be found in Supplementary Table S4.

### Scratch assay, migration and invasion assays

Cells were plated in 6-well dishes at 1 × 10 ^ 5 cells per well. The following day, a scratch was applied down each well using a 10 ul pipette tip. Images were taken using an Eclipse Ti (Nikon Instruments) on the day of the scratch and for two subsequent days. Scratch resolution was analysed using Tscratch software developed by the Koumoutsakos group (CSE Lab) at ETH Zürich^75^.

For migration assay, 2.5 × 10 ^ 4 cells were plated in serum-free media in the upper well of an 8 um pore transwell (BD); complete media was in the bottom well. Cells were fixed and stained with crystal violet after 20 hours. For assessment of invasion, transwell inserts were coated with Corning Matrigel Growth Factor Reduced (BD). 5 random 10X images were obtained from each well and used for quantification.

### Murine subcapsular model

All animal experiments were approved by the UCLA IACUC and conformed to all local and national animal care guidelines and regulations. Female 6–8 week old Nu/J mice (Jackson Laboratory) were placed in the prone position and an incision was made on the left flank^30^. The left kidney was partially exteriorized. A Hamilton syringe (28 gauge) was used to inject 1 × 10 ^ 5 cells in 5 ul of sterile PBS under the kidney capsule. Sectioning and H&E staining was done by the UCLA Pathology core. Two consecutive lung slides per animal were assessed for the number of visible tumour nodules. Peripheral blood was collected at endpoint through eye bleeding. Bioluminescent imaging was performed on an IVIS cooled CCD camera (Xenogen).

### RNA-Seq

RC and RVN cells were cultured for two days and then processed using the RNeasy Mini Kit (Qiagen) according to manufacturer’s protocols. RNA was submitted to the UCLA Clinical Microarray Core where the sequencing was performed on the Illumina HiSeq 2000 and was single read 1 × 50. Raw data was processed by the UCLA Institute for Quantitative and Computational Biosciences. The resulting data was normalized, mapped to the genome and compared between samples. The false discovery rate cut-off was set at 0.1.

### TCGA data

Data from the TCGA was queried using cBioPortal (www.cbioportal.org)^76^,^77^. All presented data utilized the Kidney Renal Clear Cell Carcinoma (TCGA, Provisional) data set.

### Human samples and ethics statement

Primary tumours and corresponding adjacent normal tissues were obtained from 45 patients who received radical nephrectomy in the Department of Urology in Tongji Hospital of Huazhong University of Science and Technology in China from January 2012 to December 2015. All patients involved consented to participate in the study before surgery and signed an informed consent form, and all experiments were performed in accordance with the approved guidelines, complying with the principles for the use of human tissues in the Declaration of Helsinki. The study was approved by the Ethics Committee of Tongji Hospital, Tongji Medical School, Huazhong University of Science and Technology.

### Statistics

Data is presented as mean +/− standard error mean (SEM). Comparisons between groups were analysed by student’s t-test.

## Additional Information

**How to cite this article**: Schokrpur, S. *et al*. CRISPR-Mediated VHL Knockout Generates an Improved Model for Metastatic Renal Cell Carcinoma. *Sci. Rep.* **6**, 29032; doi: 10.1038/srep29032 (2016).

## Supplementary Material

Supplementary Information

Supplementary Information

Supplementary Information

Supplementary Information

Supplementary Information
