# Supplementary material for: CRISPR-Mediated VHL Knockout Generates an Improved Model for Metastatic Renal Cell Carcinoma
Source: Sci Rep. 2016 Jun 30;6:29032. doi: 10.1038/srep29032 (PMC4928183; doi:10.1038/srep29032)
Supplement: Supplementary Information [file srep29032-s1.pdf]

# CRISPR-Mediated VHL Knockout Generates an Improved Model for Metastatic Renal Cell Carcinoma

Shiruyeh Schokrpur[1], Junhui Hu[1,2,3], Diana L. Moughon[1], Peijun Liu[2], Lucia C. Lin[1], Kip Hermann[1], Serghei Mangul[4], Wei Guan[1,2], Matteo Pellegrini[4,5] Hua Xu[2] and Lily Wu[1,6]

[1]Department of Molecular and Medical Pharmacology, David Geffen School of Medicine, University of California at Los Angeles CA 90095

[2]Department of Urology and Institute of Urology, Tongji Hospital, Tongji Medical College, Huazhong University of Science and Technology, Wuhan, 430030, China

[3]Department of Paediatric Surgery, Tongji Hospital, Tongji Medical College, Huazhong University of Science and Technology, Wuhan, 430030, China

[4]Department of Computer Science and Human Genetics, University of California at Los Angeles CA 90095

[5]Department of Molecular, Cell, and Developmental Biology, University of California at Los Angeles CA 90095

[6]Department of Urology, David Geffen School of Medicine, University of California at Los Angeles CA 90095

**Running Title:** CRISPR-mediated VHL loss drives metastasis in RCC

## Supplementary Methods

### Generation of non-integrated CRISPR-modified lines

Lentivirus was generated as described in Methods, except that packaging plasmid pCMV-delta-r8.2 (-INT) (kind gift from Dr. Don Kohn), which is deficient in integrase function, was used. Cells were transduced as described in Methods and treated with puromycin (2 ug/ml) from 48 hr to 96 hr following transduction. Cells were expanded and clonal selection was performed as described in Methods. Please refer to Supplementary Table 2 for details regarding ctrl (NIC) and VHLko (NIC).

### Generation of pLVX-Tight-mVHL-Puro and pLVX-Tet-Off plasmids

pLVX-Tet-Off and pLVX-Tight-Puro were a kind gift from Dr. Steven M. Dubinett. Murine VHL coding sequence was PCR amplified with forward primer (5'-CGCGGATCCGGACCCGTTCCAATAATGCC-3') and reverse primer (5'-CCGGAATTCACTCCTTCAAGGCTCCTCTTCC-3') from Renca cells' cDNA and cloned into pLVX-Tight-Puro with BamHI and EcoRI nuclease.

### Establishment of tetracycline-regulated (Tet-Off) VHL expressing cell line and rescue experiment

VHLko (NIC) cells were selected and transfected sequentially with pLVX-Tet-Off and pLVX-Tight-mVHL-Puro lentivirus followed with puromycin (1 µg/ml) and G418(500 µg/ml) selection. Doxycycline (50ng/ml) was then added to the RPMI-1640/10%FBS medium for the VHLko (NIC) tet-VHL cell line. Cell morphology was observed and RNA and proteins were extracted from VHLko (NIC) tet-VHL cells with & without doxycycline for Western blot analysis.

**HIF-2α inhibitor treatment**

VHLko (NIC) cells were cultured like other RENCA cells as described in Methods. Allosteric inhibitor of HIF-2 N-(3-chloro-5-fluorophenyl)-4-nitrobenzo[c][1,2,5]oxadiazol-5-amine (Axon Medchem 2034) was diluted in DMSO. Cells were treated with DMSO control or HIF-2α inhibitor at 10 uM. Cells were treated for 5 days with refreshed media every 24 hr before imaging. Cells were treated for 24 hr before harvesting for RT-PCR.

**Supplementary Figure S1. Guide RNAs effectively target murine VHL with varying efficiency**

a) CDS sequence of murine VHL is shown with locations of gRNA targets indicated by colour. b) RENCA cells transduced with *Renilla* targeted (upper) or VHL targeted (gRNA1, lower) lentivirus were sequenced at the VHL locus and the region which gRNA1 targets is shown. The middle sequence is the reference sequence for murine VHL. c) RENCA cells were infected with lentivirus carrying gRNAs targeting location one, location two or both location one and two on murine VHL. Lysates were collected and blotted for HIF-1α (top) and β-actin (bot.). Gene expression was analysed by RT-PCR for d) Glut-1 and e) eGFP. n=3 for RT-PCR studies

**Supplementary Figure S2. VHL loss is responsible for EMT changes**

Cells transduced with guides RLuc LC2, mVHL LC1 and mVHL LC3 underwent clonal selection following puromycin treatment. One clone from the RLuc LC2 cell line (Rc1) and two clones each from the mVHL LC1 (V1c1, V1c2) and mVHL LC3 (V3c1, V3c2) were analysed further. a) Western blot for murine VHL, HIF-1α, E-cadherin and β-actin. Gene expression was analysed by RT-PCR for b) Glut-1, c) E-cadherin d) N-cadherin and e) MMP-9. VHLko (NIC) tet-VHL cells were treated with (left) or without (right) doxycycline (50 ng/ml). f) Western blot for human VHL, HIF-1α and β-actin. g) Phase contrast 10X images. n=3 for RT-PCR studies *denotes $p<0.05$, **denotes $p<0.01$, ***denotes $p<0.001$

**Supplementary Figure S3. Human RCC line shows consistent changes of EMT with VHL loss**

ACHN human RCC cell line was treated with either RLuc gRNA or gRNAs targeting human VHL to generate ACHN ctrl, ACHN VHLko and ACHN VHLko2 cells. a) Western blot for human VHL, HIF-1α and β-actin. b) Phase contrast 10X images of (RLuc targeting) control (top) and VHL knockout ACHN cells (bot.). c-d) Gene expression was analysed by RT-PCR for E-cadherin, N-cadherin and MMP-9. *denotes $p<0.05$, **denotes $p<0.01$

**Supplementary Figure S4. Mesenchymal phenotype induced by VHL knockout is mediated by HIF-1α**

a) Western blot for murine VHL, HIF-1α and β-actin in Cn, Vko, Hko and VkoHko cells. b) Gene expression was analysed by RT-PCR for Glut-1. c) Phase contrast 10X images of Cn, Vko, Hko and VkoHko cells. d) Western blot for murine E-cadherin and β-actin. Gene expression was analysed by RT-PCR for e) E-cadherin and f) N-cadherin. n=3 for RT-PCR studies **denotes $p<0.01$, ***denotes $p<0.001$

**Supplementary Figure S5. HIF-2α is not responsible for EMT phenotype.**

a) Ratio of gene expression by RT-PCR of HIF-1α/HIF-2α is shown. b) Phenotype of cells treated with DMSO (upper) or HIF-2α inhibitor. c) Gene expression was analysed by RT-PCR for E-cadherin, N-cadherin, MMP-9, α-SMA, EPO and VEGF-A. **denotes $p<0.01$

**Supplementary Figure S6. Hypoxia mimetics recapitulate morphological changes seen upon VHL knockout but mechanism of mesenchymal transition is not through common EMT transcriptional regulators**

a) Phase contrast 10X images of wild type RENCA cells treated with 100uM $CoCl_2$ or 1mM DMOG for one week. b) Gene expression was analysed by RT-PCR for expression of Snai1, Snai2, Twist1, Zeb1 and Zeb2 in RC and RVN cells. n=3 for RT-PCR studies

**Supplementary Figure S7. 4-gene set comprised of POSTN, PPEF1, SAMSN1 and TNFSF13B is upregulated in 45 ccRCC tumour samples.**

a) POSTN, b) PPEF1, c) SAMSN1, and d) TNFSF13B expression in 45 ccRCC tumour samples compared to respective adjacent normal tissues, in which every column is the fold change "T/N" of a specific gene in a patient (T stands for tumour, N stands for corresponding adjacent normal tissue). *denotes $p<0.05$, **denotes $p<0.01$

**Supplementary Table S1. gRNA sequences targeting indicated genes**

Forward strand oligoes used for cloning into lentiCRISPR constructs are shown.

**Supplementary Table S2. List of cell lines and CRISPR constructs used to generate them**

LC represents the lentiCRISPR plasmid with puromycin selection. LCGFP represents the lentiCRISPR-eGFP plasmid in which we cloned out puromycin resistance and instead put in eGFP.

**Supplementary Table S3. Ten gene list from RNA-seq data and TCGA**

List of ten of the top 65 upregulated genes in our RNA-seq data which showed independent association with poor overall and/or progression free survival in the TCGA. Log2FoldChange is the difference in expression obtained in our RNA-seq comparing RVN to RC expression. %=

percentage of patients in the TCGA showing upregulation of these genes z=1. p value for overall survival (OS) or progression free survival (PFS) between patients with upregulation compared with no upregulation of these genes is given in the final two columns.

**Supplementary Table S4. RT-PCR primer sequences**

Forward and reverse primer sequences for genes analysed by RT-PCR are provided.

**Supplementary Video S1. RC cell video**

A phase contrast image of RC cells was taken at 10X every 15 minutes for 18hr and merged into a video.

**Supplementary Video S2. RVN cell video**

A phase contrast image of RVN cells was taken at 10X every 15 minutes for 18hr and merged into a video.

**Supplementary Video S3. RC scratch assay video**

A phase contrast image of an RC cell scratch assay was taken at 10X every 15 minutes for 18hr and merged into a video.

**Supplementary Video S4. RVN scratch assay video**

A phase contrast image of an RVN cell scratch assay was taken at 10X every 15 minutes for 18hr and merged into a video.

SUPPLEMENTARY FIGURE S1

**a**

ATGCCCCGGAAGGCAGCCAGTCCAGAGGAGGCGGCGGGGAGCCCGGTCC
TGAGGAGATGGAGGCTGGGCGGCCGCGGCCGGTGCTGCGCTCGGTGAACTC
GCGCGAGCCCTCTCAGGTCATCTTCTGCAACCGCAGCCCGCGCGTCGTGCTG
CCTTTGTGGCTCAACTTCGACGGCGAGCCTCAGCCCTACCCGATCTTACCACC
GGGCACCGGCCGCCGCATCCACAGCTACCGAGGTCATCTTTGGCTCTTCAGG
GATGCGGGGACCCATGATGGACTTCTGGTTAACCAAACGGAGCTGTTTGTGC
CATCCCTCAATGTCGATGGACAGCCTATTTTTGCCAACATCACATTGCCAGTGT
ATACCCTGAAAGAGCGGTGCCTTCAGGTTGTGCGGAGCCTGGTCAAGCCTGA
GAACTACAGGAGACTGGACATCGTCAGGTCACTCTATGAGGATTTGGAGGAC
TACCCAAGTGTGCGGAAGGACATACAGCGACTGAGCCAAGAGCACCTTGAGA
GTCAGCACCTGGAAGAGGAGCCTTGA

gRNA1 gRNA2

**b**

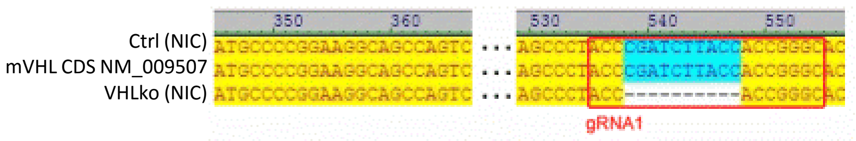


**c**

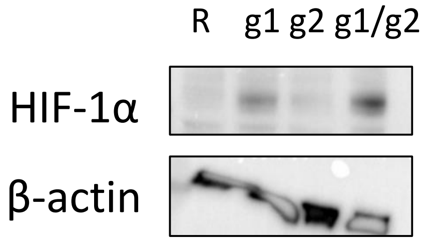


**d**

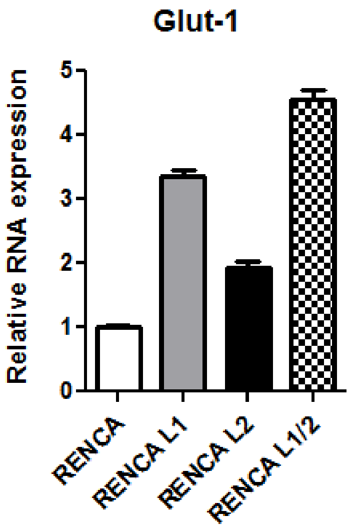


**e**

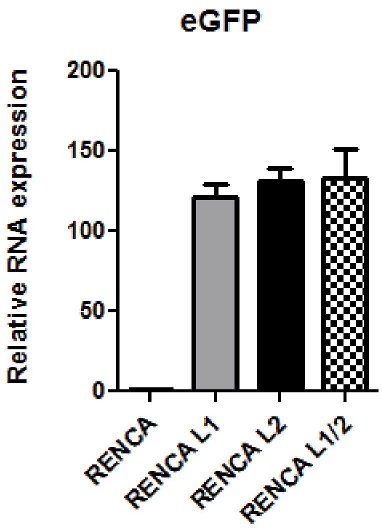

SUPPLEMENTARY FIGURE S2

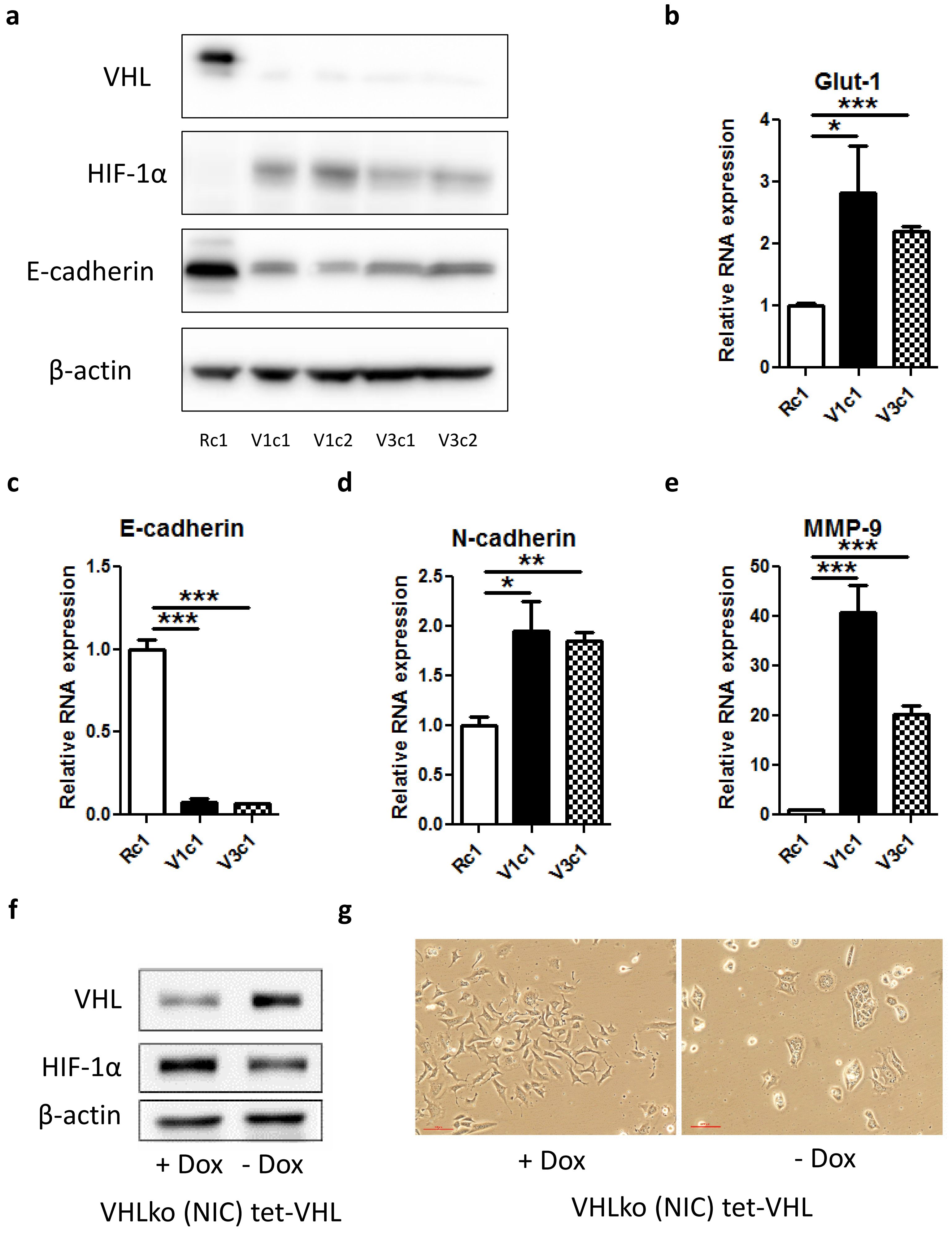

SUPPLEMENTARY FIGURE S3

**a**

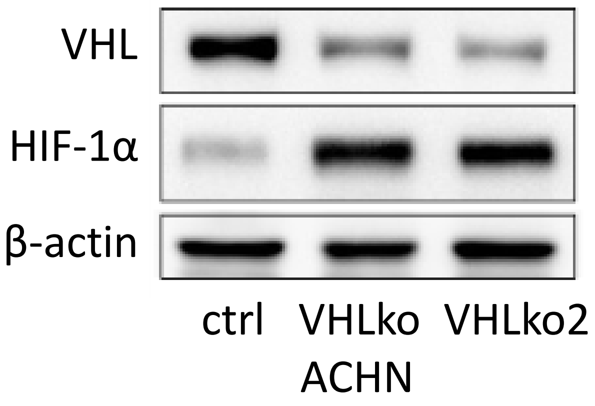


**b**

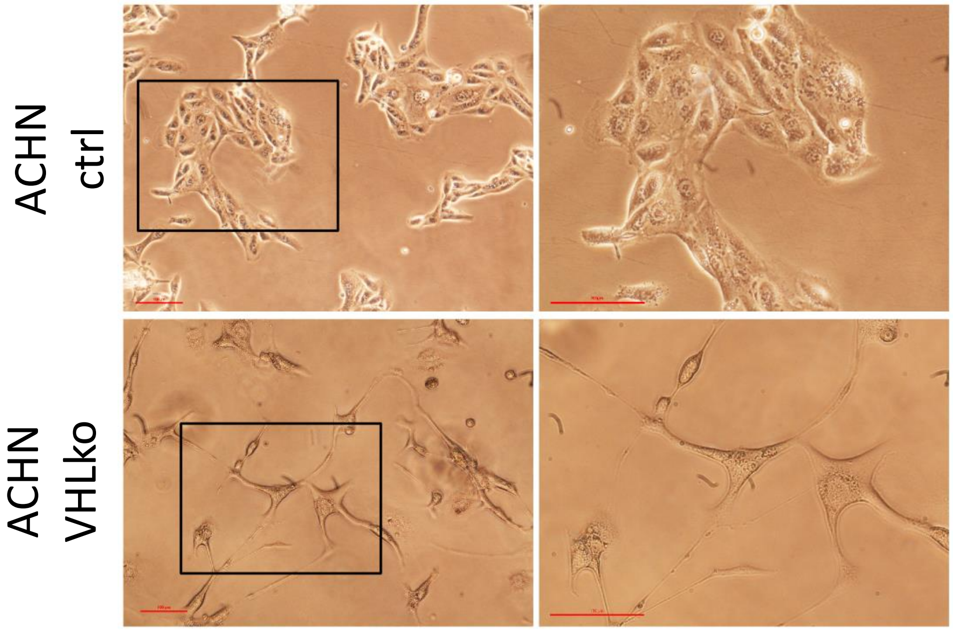


**c**

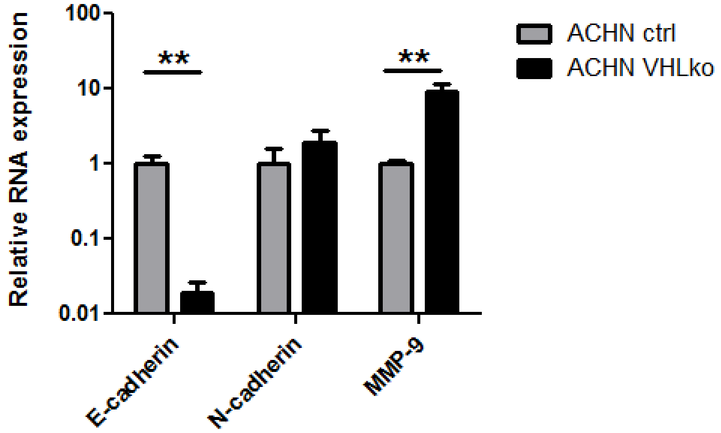


**d**

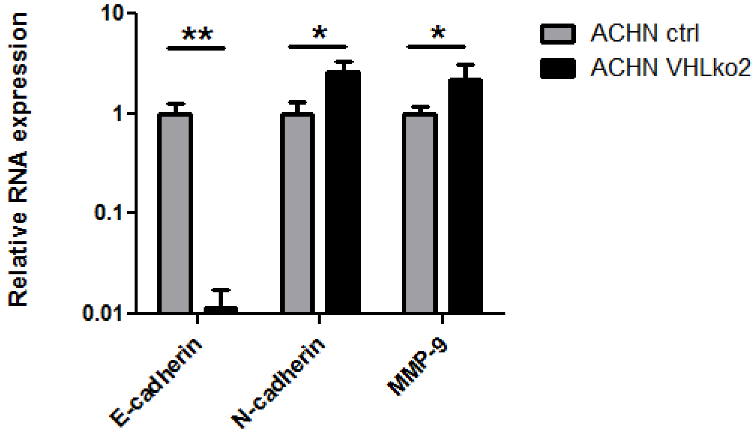

SUPPLEMENTARY FIGURE S4

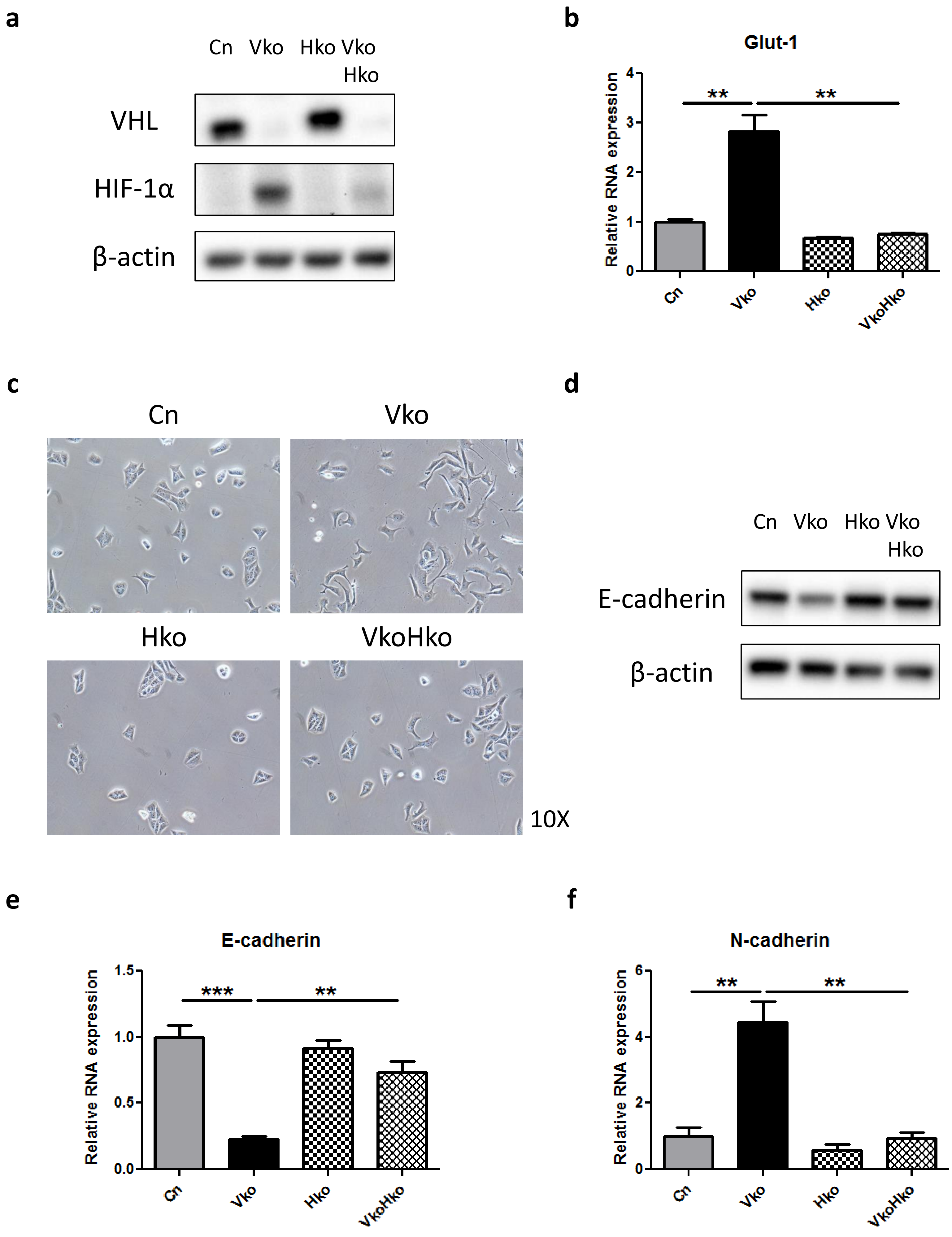

SUPPLEMENTARY FIGURE S5

**a**

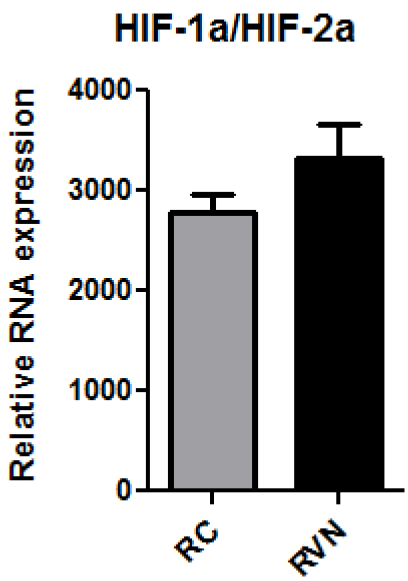


**b**

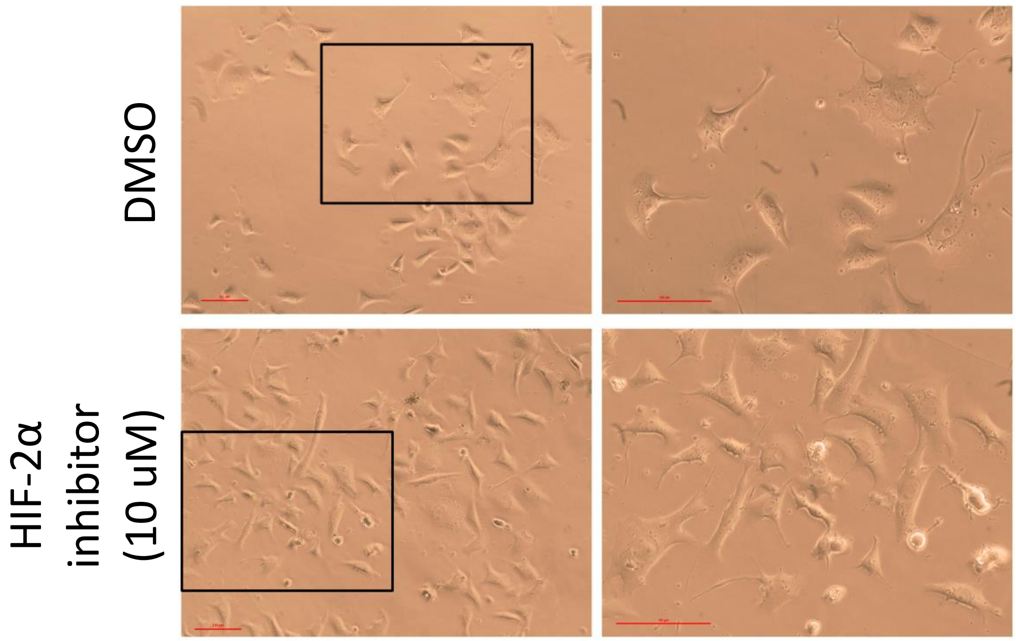


**c**

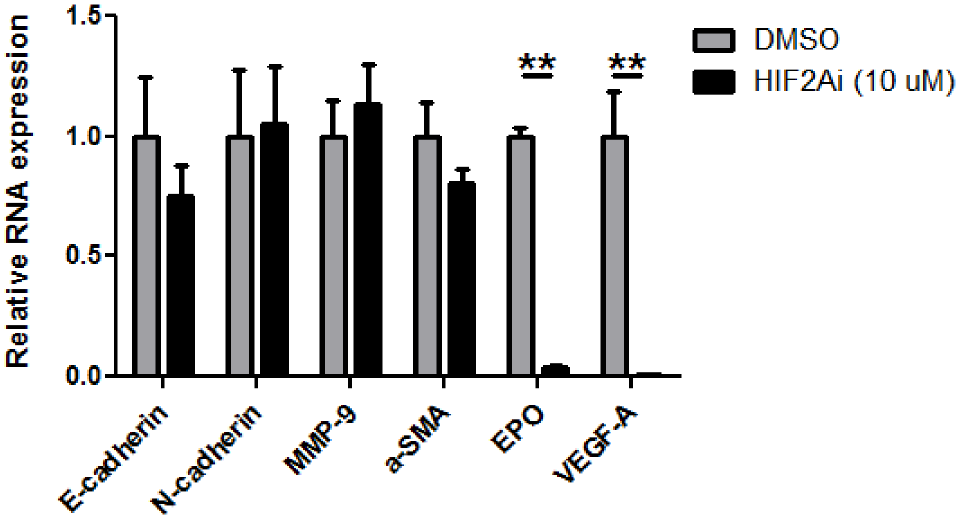

SUPPLEMENTARY FIGURE S6

**a**

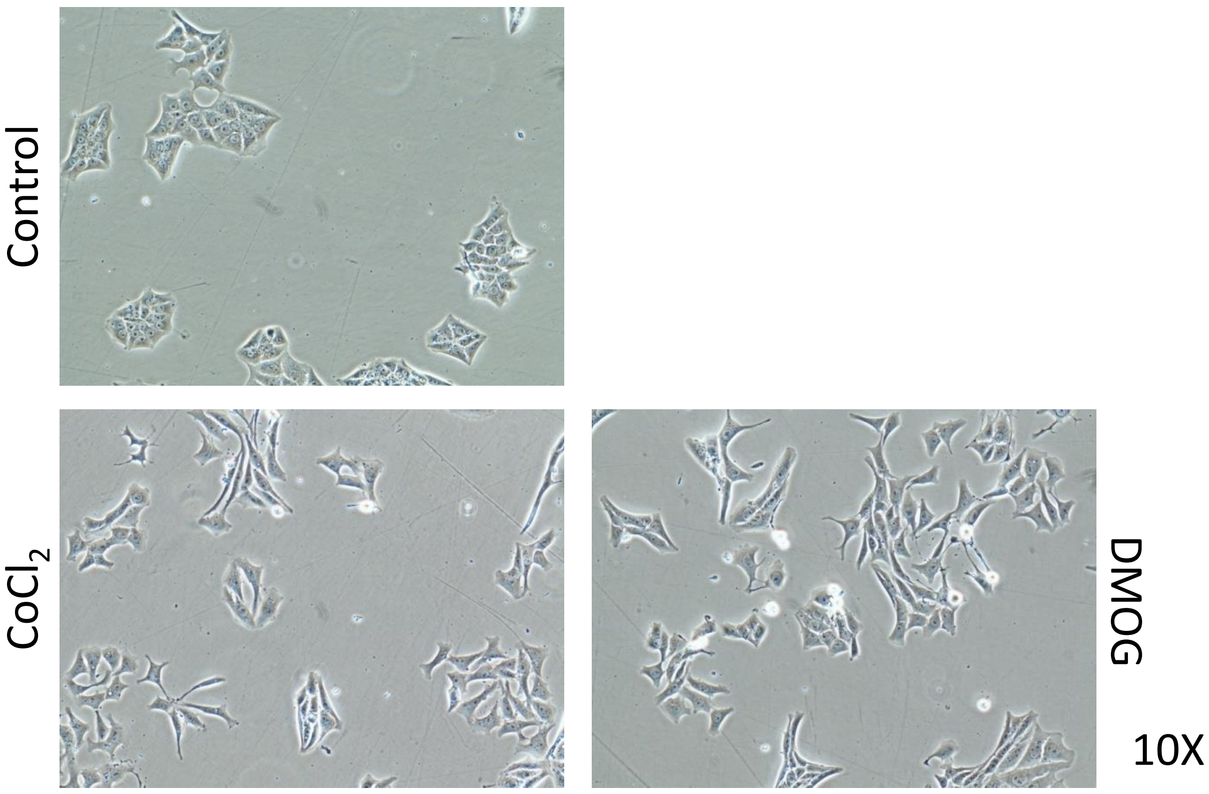


**b**

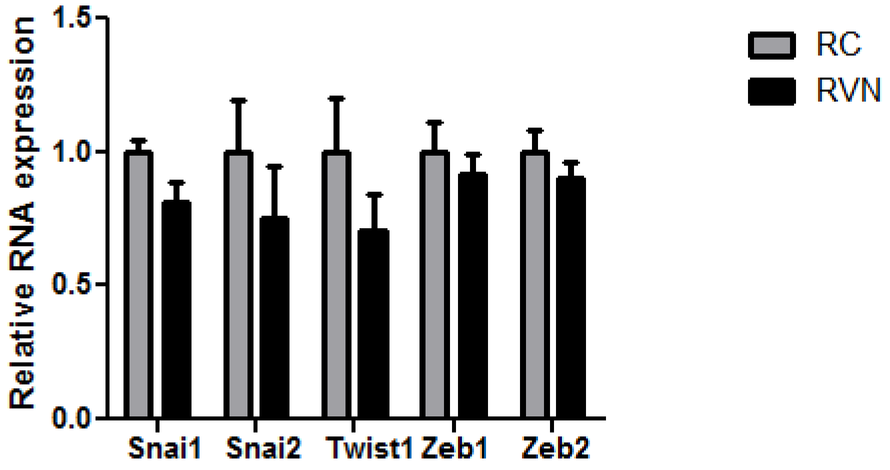

# SUPPLEMENTARY FIGURE S7

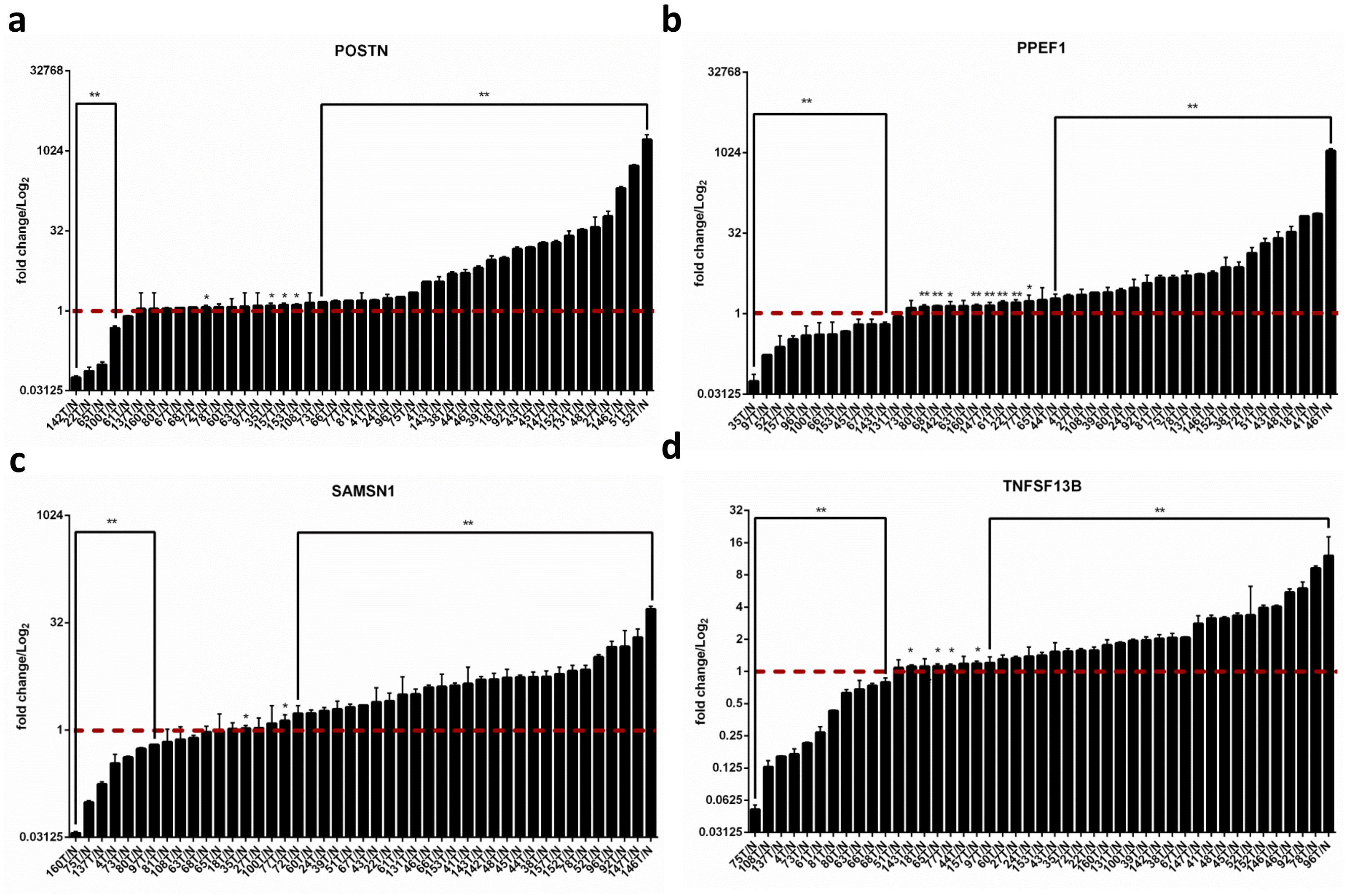

# SUPPLEMENTARY TABLE S1

| gRNA | Sequence |
|---|---|
| mVHL 1 | CACCGCCCGGTGGTAAGATCGGGT |
| mVHL 2 | CACCGAACTCGCGCGAGCCCTCTC |
| mVHL 3 | CACCGACAAAGGCAGCACGACGCGC |
| RLuc 1 | CACCGGTATAATACACCGCGCTAC |
| RLuc 2 | CACCGGATGATAACTGGTCCGCAG |
| mHIF-1α | CACCGCTAACAGATGACGGCGACA |
| hVHL 1 | CACCGAGTCCGGCCCGGAAGAGTC |
| hVHL 2 | CACCGAGGCAGGCGTCGAAGAGTA |

SUPPLEMENTARY TABLE S2

| Cell line | CRISPR 1 (plasmid) | CRISPR 2 (plasmid) | Original cell line |
|---|---|---|---|
| RVN | mVHL 1 (LCGFP) | mVHL 2 (LC) | RENCA FLuc |
| RC | RLuc 1 (LCGFP) | RLuc 2 (LC) | RENCA FLuc |
| Rc1 (Ctrl) | Rluc 2 (LC) | | RENCA |
| V1c1, V1c2 | mVHL 1 (LC) | | RENCA |
| V3c1, V3c2 | mVHL 3 (LC) | | RENCA |
| VHLko | mVHL 1 (LC) | RLuc 1 (LCGFP) | RENCA |
| VHLko/HIF1ako | mVHL 1 (LC) | mHIF-1α 1 (LCGFP) | RENCA |
| Cn | RLuc 1 (LCGFP) | RLuc 2 (LC) | RENCA FLuc |
| Vko | RLuc 1 (LCGFP) | mVHL 1 (LC) | RENCA FLuc |
| Hko | mHIF-1α (LCGFP) | RLuc 2 (LC) | RENCA FLuc |
| VkoHko | mHIF-1α (LCGFP) | mVHL 1 (LC) | RENCA Fluc |
| Ctrl (NIC) | RLuc 2 (LC) | | RENCA FLuc |
| VHLko (NIC) | mVHL 1 (LC) | | RENCA FLuc |
| ACHN ctrl | RLuc 2 (LC) | | ACHN |
| ACHN VHLko | hVHL 1 (LC) | | ACHN |
| ACHN VHLko2 | hVHL 2 (LC) | | ACHN |
| VHLko (NIC) tet-VHL | mVHL 1 (LC) | | VHLko (NIC) |

# SUPPLEMENTARY TABLE S3

| | log2FoldChange | % | OS | PFS |
|---|---|---|---|---|
| postn | 5.649907245 | 12 | 0.003729 | 0.051537 |
| col3a1 | 5.200922038 | 13 | 0.019931 | 0.003871 |
| tnfsf13b | 4.662966142 | 11 | 0.011183 | 0.060136 |
| ppef1 | 4.564576068 | 8 | 0.009865 | 0.003425 |
| adam8 | 4.373394833 | 5 | 0 | 0.012914 |
| il18rap | 4.283588417 | 10 | 0.009108 | 0.753675 |
| samsn1 | 4.074593522 | 14 | 0.00142 | 0.005059 |
| ccl7 | 4.040122704 | 5 | 0 | 0.003412 |
| cuzd1 | 4.007994747 | 7 | 0.003363 | 0.424231 |
| aif1 | 3.99434937 | 13 | 0.002657 | 0.005318 |

SUPPLEMENTARY TABLE S4

| Gene | Forward primer | Reverse primer |
|---|---|---|
| E-cadherin | CTGCTGCTCCTACTGTTTCTAC | TCTTCTTCTCCACCTCCTTCT |
| N-cadherin | AGTGGCAGGTAGCTGTAAAC | TGGCAAGTTGTCTAGGGAATAC |
| β-actin | TCAAGATCATTGCTCCTCCTGAGC | TACTCCTGCTTGCTGATCCACATC |
| Glut-1 | TCAACGAGCATCTTCGAGAAGGCA | TCGTCCAGCTCGCTCTACAACAAA |
| PGK1 | CAAGGCTGCTGTTCCAAGCATCAA | TGAGTTCAGCAGCAACTGGCTCTA |
| LDHA | ACAAACTCAAGGGCGAGATG | GGAGTTCGCAGTTACACAGTAG |
| NDRG1 | TCTTCGGCAAGGAGGAGATA | AGGCGCTGATGAATAAGTGTAG |
| α-SMA | TCAGGGAGTAATGGTTGGAATG | GGTGATGATGCCGTGTTCTA |
| MMP-9 | TGAGCTGGACAGCCAGACACTAAA | TCGCGGCAAGTCTTCAGAGTAGTT |
| Cas9 | ACCAGAAAGAGCGAGGAAAC | TCGTTGGGCAGGTTCTTATC |
| eGFP | CCACATGAAGCAGCAGGACTT | GGTGCGCTCCTGGACGTA |
| POSTN | CACGGCATGGTTATTCCTTCA | TCAGGACACGGTCAATGACAT |
| TNFSF13B | ACACTGCCCAACAATTCCTG | TCGTCTCCGTTGCGTGAAATC |
| PPEF1 | CTGACTCCTATGATGGTCCTCG | CCTCTAAGACGTAGTGAGCATGA |
| SAMSN1 | CCAAGTCCCTATGACACCGAC | CCTGGATAGTCTGGTGGTTCT |
| Snai1 | GAGAAGCCATTCTCCTGCTC | GCACTGGTATCTCTTCACATCC |
| Snai2 | CCATTAGTGACGAAGAGGAGAG | CAGCCCAGAGAACGTAGAATAG |
| Twist1 | AGCTGAGCAAGATTCAGACC | AGCTTGCCATCTTGGAGTC |
| Zeb1 | TCGGAAGACAGAGAATGGAATG | CCTCTTACCTGTGTGCTCATATT |
| Zeb2 | CTCATTCTGGGTCCTACAGTTC | GGGAAGAACCCGTCTTGATATT |
| EPO | ACTCTCCTTGCTACTGATTCCT | ATCGTGACATTTTCTGCCTCC |
| VEGF-A | GCACATAGAGAGAATGAGCTTCC | CTCCGCTCTGAACAAGGCT |
